# Over Work

**Published:** 1892-01

**Authors:** Henry Cowie


### Over Work.

BY DR. HENRY COWIE.

Read before the Detroit Dental Society. December 14th 1891.

In making selection of this topic as a subject for a short paper to be read before this association at this meeting I shall endeavor to show the way it may affect us personally as dentists, and then some of the effects of over work to be noted on the teeth, particularly on those of young girls. In considering the first part of our subject, one quite as important at least to us individually as any thing pertaining to dentistry, for it is we who have to do the work, and live by that work, and if we fail to be able to accomplish our selected mission in life through any reason such as may come from over work, it is time for us to consider if we are doing justice either to ourselves, to those depending on us, or to our patients.

I think I am justified in the belief that those of us in full practice are in great danger of so doing; the big dollar has a great attraction, an almost moving eloquence, and the most of us strive for its possession; we, perhaps not more than men in other pursuits, but it is a failure with most of us, and to attain money we all sin against the laws of health which should guide us.

One may work hard in such a way as to be called "wear" which is a natural and the legitimate result of lawful use, or we may work in such a manner, however, that it may well be called "tear" which is quite another matter, namely an abuse of our powers by over taxing our bodies and minds beyond our strength. Too long a strain will cause weakness. We can so work as to conserve our strength and thus be enabled to work a longer number of years and to do better work all the time.

Our business is one that taxes both body and mind, and combined over work of mind and body is doubly mischievous, for though a proper alternation of physical and mental labor is best fitted to insure a life-time of exertion, yet some forms of work, notably our own, bring this form of fatigue. A man who lives and works out of doors can do safely what we, who live an in-door, sedentary life, can not; he can work using his muscles, when fatigue comes he can, and generally does, warned by this feeling, take rest.

But we may not feel the effect of our muscular exertion and go on when our nervous power or force is utterly exhausted, because, unless I am mistaken, he who is using his brain sympathy, or nervous force, does not feel any particular sensation which warns him to stop taxing himself in this direction, and rest.

One is apt to get into a sort of exalted state under the influence of need, or the interest one may have in his work, and find out only in the way of headache, insomnia, eye strain, or some other of the serious forms of trouble arising from this source, that he has been doing too much—over-taxing his powers. The warning does not come always until the brain, after long suffering begins to say I have done enough.

We are apt to think our sense of being tired comes only from physical causes, arises from our constrained position, etc., when we really are quite as much, if not more tired mentally than physically. We should, therefore, try to limit our work to a certain definite number of hours so that we may take time outside of our offices for exercise and rest.

Exercise we need, but in a different form from that given by our work, and rest we must have for the over-taxed nerves, unless we are willing to accept the certain results of over work. The indications of brain tire differ. One of the usual forms is to be found when, on retiring for the night, the mind keeps turning over and over the work of the day, very often in a very disconnected way, or else the imagination soars away with the energy of a demon conjuring up processions of broken and disconnected thoughts so that sleep is utterly banished, this state of mind being fatal to sleep. Another form makes itself manifest in headaches more or less severe. These plain symptoms of distress, if unheeded, may, and very often do, result in total failure of both mind and body. But, gentlemen, each of us will decide only by personal experience, we generally try to prove every thing for ourselves before we acknowledge it; we wait until we have been round the world before we confess the world is round; we will not even too rashly accept the fact that one hundred and forty-four square inches make a square foot; in fact, we do not accept the experience of others to profit by it.

In the second part of this paper, namely, the effect of Over-Work to be noted on the Teeth, the present method of education for girls is, in my mind, responsible to a great degree for the bad condition of the teeth as we find them in too many young girls; such a condition as we may easily recall from among our young patients who are attending, or have just left school.

Teeth well-formed as to shape and position, but each tooth decayed in so many places, with soft, large, white cavities of decay, that the task of saving them permanently seems, and is, almost hopeless. These girls having been over worked by the system of study now in vogue; this over-study, over-taxing the brain and nerve power, just at an age when they are undergoing organic development, which is of itself quite enough strain, renders them very sensitive to any additional tax on their powers. In most of our schools the hours are too long, the studies are too many and particularly is this the case in our high schools. The family doctor knows how often and how earnestly he is called on to remonstrate against this evil; he is, of course, aware that many strong, sturdy girls stand the strain, but he knows just as well that many do not, that they break down and have weak eyes, headaches, neuralgia, etc., as a result of the over study.

A boy does not study as hard, that is, he will not go, as most girls do, home to study, but plays out of doors, and if he does study out of school at all he will do a little during the evening. Then, too, boys do not get the hardest part of their education until they are over eighteen, as a boy can not graduate in most of our colleges until he is twenty-one. A girl gets through, as a rule, about eighteen, so that the hardest part of her study comes just during the time when she should not be so over-taxed.

We, of course, are principally interested in the condition of their teeth, but we also know that if the general health is bad, and the development is checked, that the condition will be of the worst, so far as the condition of the teeth is concerned.

---

Doctors and Politics.—It is not generally known, says the *San Francisco Chronicle*, that Marat, the revolutionist dispatched by Charlotte Corday, was a physician, and that he had a certain success in treating consumption. It is recorded of him that he cured a titled lady of this disease in its advanced stages, and that her gratitude to him knew no bounds. Unfortunately he was enticed into politics and prevented from pursuing his studies further in a direction that might have made his memory revered instead of detested.

For some reason doctors seem to drift naturally toward politics and radicalism, perhaps because their profession tends to render them skeptical. In Brazil and the Argentine Republic they have shown themselves decidedly ambitious. The celebrated Dr. Charcot is a radical whose principles verge on the revolutionary. Clemenceau gave up his medical practice years ago to devote himself to politics, and though he is a man of great talent and a brilliant orator, his political efforts have not contributed to his personal advancement or been of great benefit to his country. Besides Clemenceau there are from forty to fifty physicians in the French Chamber of Deputies.

